# Uncovering superior alleles and genetic loci for yield-related traits in mungbean (*Vigna radiata* L. Wilczek) through a genome-wide association study

**DOI:** 10.1186/s12870-026-08973-1

**Published:** 2026-05-19

**Authors:** Md Shahin Uz Zaman, Md Shahin Iqbal, Md Golam Azam, Md Jahangir Alam, M. Asaduzzaman Prodhan, Md Sultan Mia, William Erskine, A.K.M. Mahbubul Alam, Ramakrishnan Madhavan Nair

**Affiliations:** 1 Pulses Research Centre, Bangladesh Agricultural Research Institute, Ishwardi-6620, Pabna, Bangladesh; 2 College of Agricultural Sciences, IUBAT-International University of Business Agriculture and Technology, Uttara Model Town, Dhaka, 1230 Bangladesh; 3 School of Biological Sciences, The University of Western Australia, 35 Stirling Highway, Perth, WA 6009 Australia; 4 School of Agriculture and Environment, The University of Western Australia, 35 Stirling Highway, Perth, WA 6009 Australia; 5 Pulses Research Centre, Regional Agricultural Research Station, Bangladesh Agricultural Research Institute, Hatahazari-4330, Chattogram, Bangladesh; 6 World Vegetable Center, South Asia /Central Asia, ICRISAT Campus, Patancheru, 502324 Hyderabad, Telangana India

**Keywords:** Mungbean, Phenology, Yield related traits, GWAS, Favorable alleles, Genomic prediction

## Abstract

**Background:**

Mungbean is a key warm-season legume crop in South and Southeast Asia, but its low productivity, driven by limited genetic diversity, necessitates dissecting the phenotypic diversity and genetic basis of yield-related traits to develop stable, high-yielding varieties.

**Results:**

In this study, 296 mungbean germplasm from the World Vegetable Center mini-core collection were evaluated in Bangladesh. Of these, 206 genotypes produced flowers and seeds, and these were subsequently evaluated over three years. These genotypes exhibited significant variation in phenological and yield-related traits: flowering time, maturity, plant height, pods per plant, 100 seeds weight and seed yield. Moderate to high broad−sense heritability was found for all phenotypic traits. The significant environmental (year) effects and genotype × year interaction, and comparatively lower heritability for the combined multi-year (MET) analysis compared to single-year analysis for most of the traits highlighted strong environmental influences. Using MET data, a genome-wide association study (GWAS) using 4,307 high quality SNPs obtained from DArT sequencing identified 18 significant SNPs located in 16 genomic regions across the six mungbean chromosomes (1, 2, 5, 6, 7 and 8) associated with the six traits. Further, we identified five genotypes (G91, G106, G107, G125, and G130) with a higher number of favorable alleles and superior yield performance. We also employed genomic prediction models and found moderate prediction accuracies (> 30%) for 100 seeds weight and seed yield.

**Conclusions:**

This study has identified a few promising genotypes and several novel genomic regions and putative candidate genes. These results will assist in incorporating important alleles into elite mungbean germplasm through marker-assisted breeding and/or genomic prediction to improve mungbean yield. Future studies should validate these loci and genotypes across diverse environments and breeding populations to ensure their stability and practical usefulness in cultivar development.

**Supplementary Information:**

The online version contains supplementary material available at 10.1186/s12870-026-08973-1.

## Introduction

Mungbean [*Vigna radiata* (L.) R. Wilczek var. *radiata*] is one of the most significant legume crops grown globally due to its nutritional, agronomic and economic benefits. Currently, mungbean is cultivated on approximately 7.3 million hectares worldwide, with an average yield of 860 kg ha^− 1^ [1]. The grains are rich in easily digestible dietary protein (20–32%), carbohydrate (53.3–67.1%), lipids (0.71–1.85%), vitamins, minerals, fiber, and beneficial phytonutrients [2], making them an essential component of balanced, cereal-based diets. Additionally, mungbean contributes to soil fertility by fixing atmospheric nitrogen in association with native rhizobia [3], which decreases demand for nitrogen fertilizer. Its role in cropping systems enhances soil health and sustainability.

Mungbean originates from the Indian subcontinent [4] and has been widely adopted in various parts of the world, including Australia and East Africa due to its high nutritional value, adaptability to diverse climatic conditions, and dual-purpose use as both food and fodder. In South and Southeast Asia, it is an essential component of rice-based cropping systems due to its relatively short growth cycle of 55–75 days [5]. In Bangladesh, mungbean is the most widely grown pulse crop (33% of total pulse cultivation), leading in both cultivation area (2.24 lakh hectares) and total production (2.78 lakh metric tons). Despite its potential as a valuable crop, productivity remains a challenge, primarily due to low seed yield, emphasizing the need for improvements in breeding, agronomic practices, and stress tolerance mechanisms to enhance overall productivity and ensure better returns for farmers [5, 6]. Therefore, improving seed yield is the main goal in its breeding. Understanding the genetics and genomics of key phenological and yield-associated agronomic traits, such as seed size, seed number and pod number is essential for effectively incorporating these traits into elite varieties. This knowledge plays a crucial role in breeding for enhanced crop productivity and resilience. Quantitative trait loci (QTLs) for agronomic traits have been identified in various crops, including wheat [7], rice [8], corn [9], common beans [10], and black gram [11]. In mungbean, several QTLs for agronomic traits have been mapped using SSR markers [12]. However, conventional linkage mapping relies on structured crosses between genetically distinct parents capturing only a limited portion of phenotypic variation. Moreover, its resolution is constrained by recombination events [13]. These limitations highlight the need for more advanced genomic approaches to enhance mungbean breeding efforts.

In recent years, genome-wide association studies (GWAS) have emerged as a powerful approach for dissecting complex traits, offering higher mapping resolution than traditional biparental mapping. GWAS leverages natural genetic variation and historical recombination events in diverse germplasm panels, relying on linkage disequilibrium (LD) between single nucleotide polymorphisms (SNPs) and quantitative trait loci (QTLs) for trait association [14]. The efficiency of GWAS is largely influenced by the extent of LD decay, which determines the resolution of identified loci. In cultivated mungbean, LD extends between 72 and 436 kb, whereas in wild mungbean, it ranges from 3 to 60 kb [15–18]. Advances in genotyping technologies such as next-generation sequencing (NGS), SNP arrays, and genotyping-by-sequencing (GBS), combined with robust bioinformatics tools, have further enhanced the precision and applicability of GWAS in crop improvement. GWAS has been successfully applied in various legume species, including soybean (*Glycine max* L.) [19], pigeon pea (*Cajanus cajan* L.) [20], common bean (*Phaseolus vulgaris* L.) [21], chickpea (*Cicer arietinum* L.) [22], red clover (*Trifolium pratense* L.) [23] and *Medicago truncatula* [24]. In mungbean, although GWAS studies are relatively few, they have identified candidate genes linked to key agronomic and stress-related traits such as time to flowering [25]; time to maturity [26]; seed size [27]; waterlogging tolerance [28]; salinity [18]; and resistance to Yellow Mosaic Virus in Vigna Species (MYMV) [29].

While GWAS is effective for detecting significant genetic associations, it often misses the effects of rare variants, limiting its use in conventional marker-assisted selection [14]. Genomic prediction (GP) is another genomic-based breeding approach that predicts an individual’s phenotypic performance using genomic data. Unlike marker-assisted selection, which relies on a few markers, GP uses genome-wide genotypic data to estimate genetic values (genomic estimated breeding values, GEBVs) without requiring phenotypic measurements [30]. This allows GP to capture minor-effect QTLs, making it a powerful tool for accelerating genetic gains in complex traits such as yield within a short time frame [31, 32]. GP has been successfully applied in different grain legumes [33, 34]. Recently, Iqbal et al. [18] demonstrated the potential of using GP for enhancing salinity tolerance in mungbean. However, its potential for phenological and yield contributing traits in mungbean breeding remains largely unexplored. In this study, a diverse panel of mungbean mini-core germplasm was evaluated over three years, and a genome-wide association study (GWAS) was performed for key phenology and yield-associated traits using DArTseq-derived SNP markers. Specifically, we aimed to (i) assess phenotypic diversity and local adaptation of World Vegetable Center’s mungbean mini-core germplasm in Bangladesh, (ii) identify novel genomic regions associated with phenology, seed yield, and related traits, and (iii) explore the potential of genomic prediction for improving yield contributing traits in mungbean. Our findings will provide a valuable genomic resource for understanding key phenological and yield contributing traits for advancing mungbean improvement.

## Materials and methods

### Plant material and field trials

A total of 296 genotypes of mungbean from the World Vegetable Center [35] mini-core germplasm collection were grown in the Spring season from March to May 2016 for initial evaluation at the Pulses Research Centre, Bangladesh Agricultural Research Institute (BARI), Ishwardi (24°9′N; 89°4′E; 19 m a.s.l.), Pabna, Bangladesh. Only 206 genotypes produced flowers and pods. These 206 genotypes represented diverse geographical origins, with the majority coming from South Asia (151), followed by Southwest Asia (21), and South East Asia (17), East Asia (4), North America (5), Europe (2), the Oceania-Pacific (3), United Kingdom (2) and Africa (1) (Supplementary Table S1). These 206 genotypes were then re-evaluated over three consecutive years: 2017, 2018, and 2019, along with two local check varieties during the spring season (March-May). The phenotypic evaluation was conducted in an alpha lattice design (13 × 16) with two replications in each year. The genotypes were planted in two rows, each 2 m long, with a 10 cm plant-to-plant spacing and a 40 cm row-to-row spacing. The average temperature in the experimental field ranged from 35 ± 7 °C during the day to 26 ± 4 °C at night during the experimental period from March to May, with field temperatures varying from a minimum of 18 °C to a maximum of 42 °C across the years.

### Phenotypic evaluation and statistical analysis

Data were collected from five randomly selected plants per genotype in each plot for traits including plant height at 90% pod maturity, number of pods per plant, 100 seeds weight (g), and seed yield (g plot⁻¹). Time to 50% flowering (days) and 90% pod maturity (days) were recorded at the plot level. Phenotypic data were first analyzed separately for each year (2017, 2018, and 2019) to explore the genotypic variation within the trial, followed by a multi-year (MET) analysis to assess the overall genotypic performance while accounting for genotype × year interactions. Descriptive statistics were generated using Meta-R v6.0 [36], and further statistical analyses for single year- and multi-year (MET) data were performed with the “lme4” package [37]. 

The linear model for analyzing the data of the single year and multi-year (MET) for alpha lattice design was done using the formula:

$$\begin{aligned}Y_{ijk} &= \mu + \mathrm{Rep}_i + \mathrm{Block}_j(\mathrm{Rep}_i) + \mathrm{Gen}_k \\&+ \varepsilon_{ijk}\,\mathrm{(across \, replicates, within\,the\, single\,year)}\end{aligned}$$

$$\begin{aligned}\mathrm{Y_ijk1}& = \mu + Year_1 + Rep_i(Year_1) + Block_j(Year_1 Rep_i) + Gen_k \\&+ Gen_k \times Year_1+\epsilon_{ijk1}\mathrm{(across\, replicates,across\,multi-year)}\end{aligned}$$

Where Y_ijk_ and Y _ijkl_ represent the trait of interest, µ is the overall mean effect, Rep_i_ is the effect of i^th^ replicate, Block_j_ (Rep_i_) is the effect of j^th^ incomplete block within the i^th^ replicate, Gen k is the effect of the k^th^ genotype and εijk is the error effect associated with the i^th^ replication, j^th^ incomplete block and k^th^ genotype, assumed to be normally distributed with zero mean and variance σ2ε [36]. Year_l_ and Gen_l_ × Year_i_ are the effects of the l^th^ year and Genotype × Year (G × Y) interactions represented by the effect on the i^th^ genotype in the l^th^ year in the linear model for integrated analysis for multi-year (MET). The genotype is considered as random effects for the single year analysis and the genotypes, year and genotype × year are considered as random effects for multi-year (MET) analysis. The resulting analysis produced the adjusted trait phenotypic values as BLUPs (Best linear unbiased predictions) for single year and multi-year. The broad-sense heritability of traits in single year and multi-year (MET) was calculated as:

$$\:{{H}}^{2}=\:\frac{{\upsigma\:}2\:{g}\:}{{\upsigma\:}2\:{g}\:+\frac{{\upsigma\:}2\:\mathrm{e}\:}{{n}\:{r}\mathrm{e}{p}{s}}\:}\:{f}\mathrm{o}\mathrm{r}\:\mathrm{s}\mathrm{i}\mathrm{n}\mathrm{g}\mathrm{l}\mathrm{e}\:\mathrm{y}\mathrm{e}\mathrm{a}\mathrm{r}\:\mathrm{a}\mathrm{n}\mathrm{a}\mathrm{l}\mathrm{y}\mathrm{s}\mathrm{i}\mathrm{s}$$

$$\:{{H}}^{2}=\:\frac{{\upsigma\:}2\:{g}\:}{{\upsigma\:}2\:{g}\:+\frac{{\upsigma\:}2\:{g}{y}\:}{{n}\:\mathrm{y}\mathrm{e}\mathrm{a}\mathrm{r}}\:+\frac{{\upsigma\:}2\:{e}}{\mathrm{n}\:{y}{e}{a}{r}\:\times\:{n}\:{r}\mathrm{e}\mathrm{p}\mathrm{s}}\:}\:{f}{o}{r}\:{m}{u}{l}{t}{i}-{y}{e}{a}{r}\:\left({M}{E}{T}\right)\:{a}{n}{a}{l}{y}{s}{i}{s}$$

Where σ^2^g and σ^2^e are the genotype and error variance components, respectively, σ^2^gy is genotype by year interaction variance, n year is the number of years, and n reps is the number of replicates [36].

To examine the relationship among all the traits studied in this study, a principal component analysis (PCA) was performed using the single year derived BLUP for each genotype in each year of 2017, 2018 and 2019 to explore the relationship among traits across years. Later, another PCA was performed for the studied six traits using the multi-year (MET) derived BLUP for each genotype to explore the overall relationship between the traits.

### Genotyping and linkage disequilibrium (LD)

A total of 24,870 SNPS obtained from DArT sequencing approach (DArTseq) at Diversity Arrays Technology (DArT P/L, Australia) were accessed from the World Vegetable Center [38]. SNPs with missing chromosome position were removed and following stringent filtering criteria (minor allele frequency ≥ 5% and call rate ≥ 50%) in TASSEL software, 4,307 high-quality SNPs were selected for further analysis. Linkage disequilibrium (LD) among marker datasets was assessed by calculating the squared allele frequency correlation (r²) between SNP marker pairs using a sliding window of 50 markers in TASSEL. Genome-wide LD decay was examined by plotting the average r² values against the physical positions of SNPs in R 4.2.2. A locally weighted polynomial regression (LOWESS) curve was fitted to visualize LD decay, with the decay distance determined at the point where the average pairwise r² declined to half of its maximum value.

### Population structure and genetic diversity analysis

Population structure and genetic diversity were analyzed using a comprehensive marker dataset. Principal Component Analysis (PCA) was performed using the Genomic Association and Prediction Integrated Tool (GAPIT) version 3 [39], and the PCA plot was visualized with the ggplot2 package in R 4.2.2. To determine the optimal number of principal components (PCs) for capturing population structure, the scree plot generated by GAPIT was examined, and the elbow point was used to select the appropriate number of PCs [40]. Further analysis of population stratification was conducted using the STRUCTURE software [41]. Shared ancestry patterns were evaluated by testing K values ranging from 1 to 10, with each value being repeated three times. Individuals with a family relationship coefficient (Q value) greater than 70% were classified into distinct subgroups, while those with lower values were considered admixed [38]. Additionally, a neighbor-joining dendrogram was constructed based on genetic distance estimates from the kinship matrix output of GAPIT [42]. The geographical origins of the 206 mini-core germplasm were represented by color codes incorporated into the dendrogram.

### Genome-wide association study (GWAS)

Genome-wide association studies (GWAS) were conducted using only the multi-year (MET) derived BLUPs of 206 genotypes for the six yield-related phenotypic traits, using 4,307 high-quality filtered SNPs in the R package Genomic Association and Prediction Integrated Tool (GAPIT), version 3 [39]. The analysis incorporated five statistical models: (i) the general linear model (GLM) [43], (ii) the mixed linear model (MLM) [44], (iii) the compressed MLM (CMLM) [45], (iv) the fixed and random model circulating probability unification (FarmCPU) [46], and (v) the Bayesian-information and Linkage Disequilibrium Iteratively Nested Keyway (BLINK) [47]. The most suitable GWAS statistical model was chosen based on the evaluation of Q-Q plots and Manhattan plots to mitigate P-value inflation. BLINK was selected as the most appropriate model due to its minimal evidence of P-value inflation. The Bonferroni correction threshold − log10 (*p*) > 4.93 (*p* = 0.05/N; N = total markers used) was overly stringent in this study, given the moderate marker density and the extent of linkage disequilibrium in mungbean. Therefore, less strict *p*-value thresholds of − log10 (*p*) > 3 were set for the identification of true marker-trait association [18]. The phenotypic variation explained (PVE) by each significant SNP was calculated as the squared correlation between phenotype and genotype [48]. Manhattan plots were generated using the ‘qqman’ package [49] in R 4.2.2. Significantly associated SNPs and their corresponding candidate genes were analyzed within the mungbean reference genome assembly [50]. The nearest neighboring genes within the LD decay (334 kb) upstream and downstream of each significant SNP were identified as positional candidate genes [18].

### Genomic prediction

The genomic prediction was explored for only the multi-year (MET) derived BLUPs of each trait using the ridge regression best linear unbiased prediction (rrBLUP) and genomic best linear unbiased prediction (GBLUP) based on the mixed-model: y = Xβ + Zµ + ε, where β and µ represent the vectors of fixed and random effects, respectively, and ε is the residual error. To validate the genomic prediction accuracy, the dataset was randomly divided into training and testing sets at 80 and 20% respectively. To manage the challenges of overfitting, the cross-validation was conducted in five hundred cycles of iterations. The predictive ability was estimated as the Pearson’s correlation coefficient between the observed and predicted phenotypic values of the test set based on the effect estimates of germplasm in the training set. The models were implemented using the “rrBLUP” package [51] in the R environment.

## Results

### Phenotypic evaluation

The variance component analyses for the single year revealed significant genotypic differences for all studied traits, except 100 seeds weight, across the three years, highlighting substantial genetic variability among the genotypes. In the single-year analysis, broad-sense heritability was generally high, particularly for seed yield (0.86–0.94), plant height (0.74–0.92), and time to flowering (0.73–0.88), indicating a strong genetic influence on these traits (Table 1). Despite the high heritability, the non-significant genotypic differences in 100 seeds weight may be due to low genetic variance relative to experimental error. The multi-year (MET) variance component analysis showed that genotype, year, and genotype × year interactions significantly affected most traits, except seed weight, which remained non-significant, indicating strong environmental influence and differential genotypic responses across years (Table 2). For traits, plant height and pods plant^− 1^, the amount of total variance was largely dominated by the environmental effects, whereas seed yield was the most influenced by the genotype × environment interaction. Broad-sense heritability estimates for the MET analysis were moderate for flowering (0.72) and maturity (0.59), whereas lower values were observed for pods per plant (0.12) and seed yield (0.45), reflecting the stronger impact of environment and G×E interaction on these traits.

**Fig. 1:**
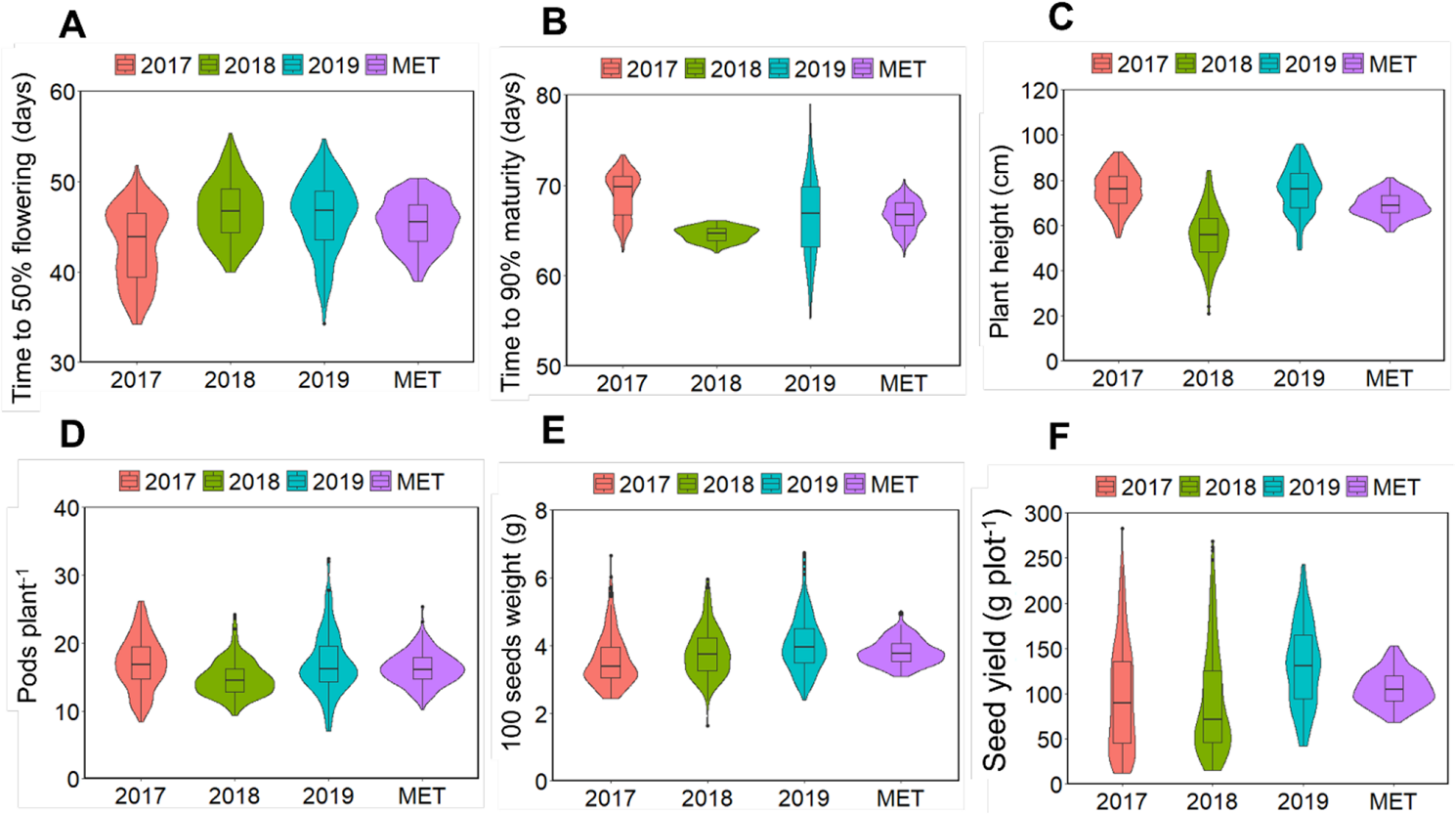
Violin plots with embedded boxplots depicting the distribution of the BLUP values for the 206 genotypes across the years 2017, 2018, and 2019, along with the combined multi-year (MET) derived BLUPs for six yield-related traits (**A**-**F**). The violin plots illustrate the data density, where wider sections indicate higher concentrations of values, while the overlaid boxplots highlight the median, interquartile range, and outliers of the BLUPs

The violin plots with embedded boxplots illustrated both the spread and density of BLUP values of the six traits (Fig. 1A–F). The distribution of the trait values demonstrated distinct year-to-year fluctuation in phenology, plant height and yield traits, indicating year-specific environmental effects. For instance, overall genotypes flowered earlier in 2017 compared with 2018 and 2019, whereas they matured earlier in 2018 than in the other two years. Plant height showed greater interannual variability, with a significant reduction in 2018 relative to the other years, possibly due to climatic differences during the growth. The pods plant^− 1^ and 100 seeds weight showed moderate variation among the years, whereas seed yield displayed the widest differences among the years, indicating a strong influence of environmental factors and G×E interactions on productivity. The single-year derived BLUPs showed wider value ranges for most traits across different years, reflecting greater variability among the genotypes. In contrast, the MET-derived BLUPs showed narrower distributions for all traits compared with single-year data, highlighting the reduction of environmental noise and producing overall stable genotypic performance across years. The violin plots for the MET data also showed frequency distributions, where all traits displayed approximately symmetrical, bell-shaped distributions, suggesting a near-normal frequency pattern indicating polygenic, quantitatively inherited traits.

**Fig. 2:**
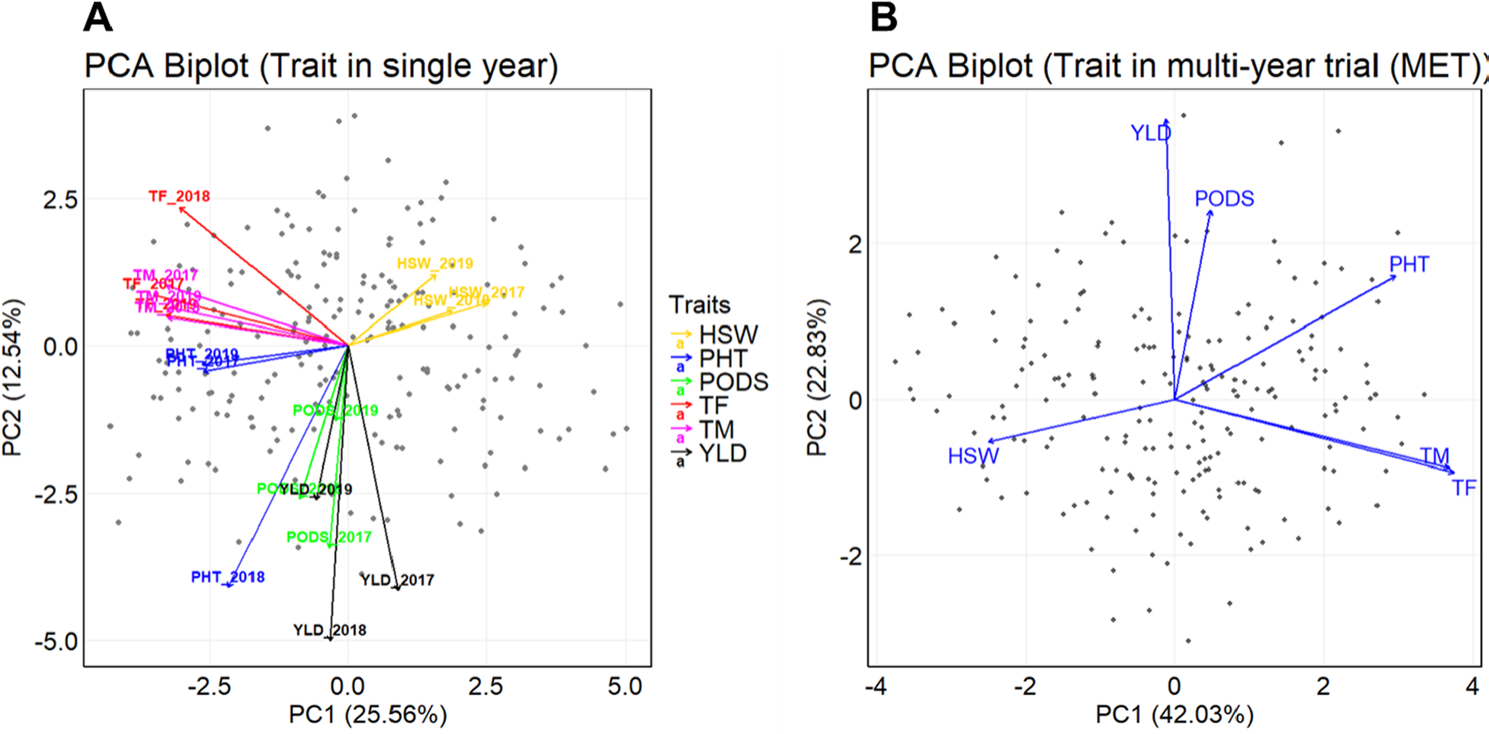
**A** Relationships among the traits - time to 50% flowering (TF), time to 90% maturity (TM), plant height (PHT), pods plant^− 1^ (PODS), 100 seeds weight (HSW) and seed yield (YLD) across the three years 2017, 2018 and 2019 of the 206 mungbean genotypes. Principal component (PC) analysis was performed using best linear unbiased predictors (BLUPs) for each trait in each year. **B** Relationship among different traits where PCA analysis was performed using the BLUPs derived from the combined multi-year (MET) analysis

Furthermore, the PCA biplots exhibited a comprehensive view of trait interrelationships across three environments (2017, 2018, and 2019) and multi-year (MET) analysis (Fig. 2). In the single-year PCA analysis, the first two principal components (PC1 and PC2) together explained 39% of the total variation (26% and 13%, respectively). The biplot shows that seed yield (YLD) consistently clustered with pods plant^− 1^ (PODS) across all years, indicating a strong positive association between these two traits. In contrast, time to flowering (TF) and maturity (TM) were closely grouped but oriented in the opposite direction to yield-related traits, reflecting a negative association between phenological traits and yield (Fig. 2A). Plant height (PHT) in 2018 was moderately aligned with pods plant^− 1^ and seed yield, implying their positive contribution to productivity in that environment. This PCA biplot also clearly demonstrated pronounced environmental effects for most traits, especially for plant height in 2018, which was distinctly separated from the other two years, suggesting strong environmental fluctuations in 2018 (Fig. 2A). In contrast, the combined multi-year (MET) PCA analysis explained a larger proportion of the total variation, with PC1 and PC2 accounting for 62% (37% and 25%, respectively), indicating a stronger underlying structure after accounting for environmental variation (Fig. 2B). This plot again clearly highlighted a strong positive association between seed yield and pods plant^− 1^ along with a moderate association with plant height and a negative association with both time to flowering and maturity (Fig. 2B).

**Table 1:** Variance component analysis of the six traits and broad-sense heritability analyzed for each year of 2017, 2018, and 2019

Traits	2017		2018		2019	
	Genotypic variance	Broad-sense heritability	Genotypic variance	Broad-sense heritability	Genotypic variance	Broad-sense heritability
Time to 50% flowering	19.03***	0.88	14.75***	0.73	16.99***	0.83
Time to 90% maturity	7.63***	0.79	1.51**	0.44	23.93***	0.83
Plant height (cm)	88.33***	0.79	150.90***	0.74	106.73***	0.92
Pods plant^− 1^	19.80***	0.75	10.26***	0.68	593.80***	0.89
100 seeds weight (g)	0.62^n.s^.	0.96	0.56 ^n.s^.	0.93	0.48 ^n.s^.	0.93
Seed yield (g plot^− 1^)	3709.90***	0.94	4022.00***	0.86	2314.60***	0.90

*** and ** represents the significance level at *P* < 0.001 and *P* < 0.01 and n.s.= non-significant

**Table 2:** Variance component analysis of the six traits and broad-sense heritability for the combined multi-year (MET) analysis

Traits	Variance				Broad-sense heritability
	Genotype	Year	Genotype × Year	Error	
Time to 50% flowering	9.96***	4.23***	7.54***	7.60	0.72
Time to 90% maturity	4.62***	4.63***	6.61***	5.78	0.59
Plant height (cm)	44.8***	133.54***	70.50***	35.08	0.59
Pods plant^− 1^	10.03***	1593.14***	201.12***	53.56	0.12
100 seeds weight (g)	0.22 ^n.s^.	0.06^n.s^.	0.41^n.s^.	0.07	0.62
Seed yield (g plot^− 1^)	795.40***	449.80***	2589.20***	682.50	0.45

*** represents the significance level at *P* < 0.01 and n.s.= non-significant

### SNP calling

**Fig. 3:**
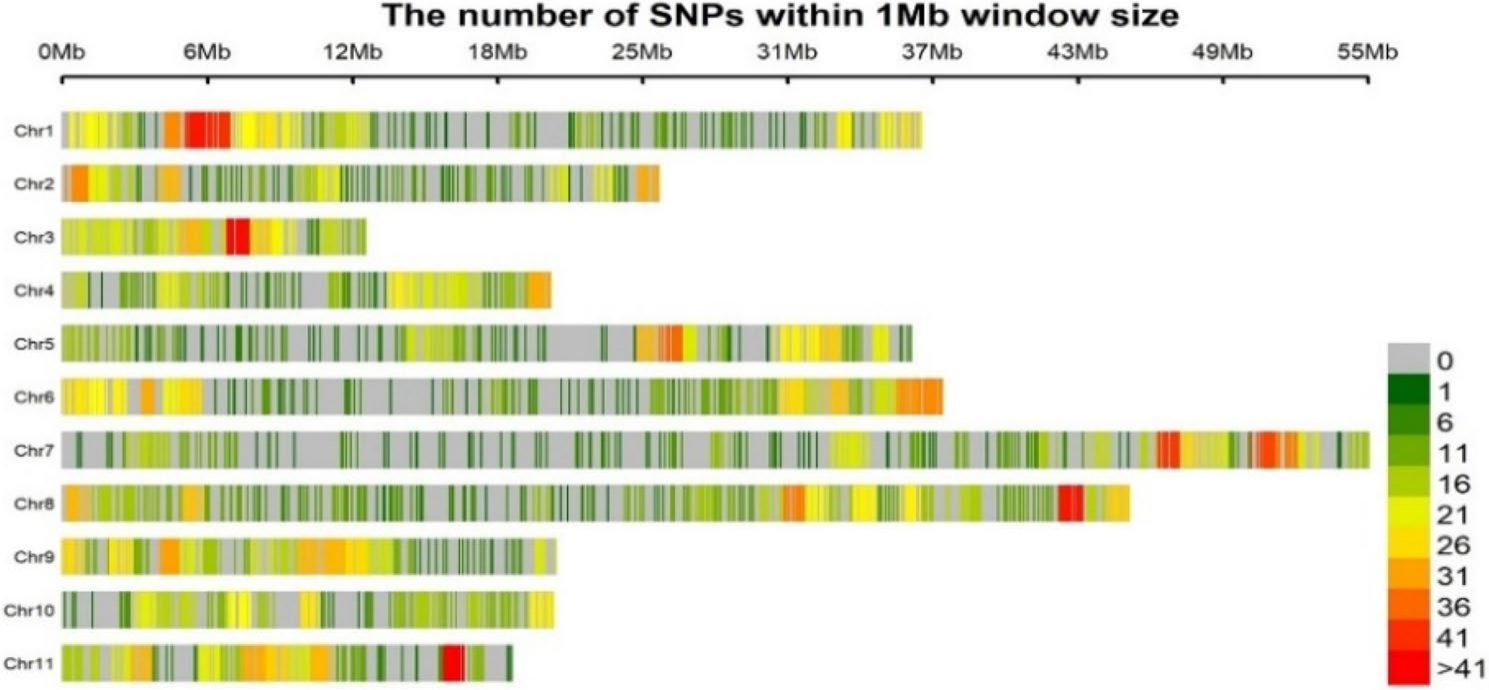
Physical map of 4,307 SNPs identified in 206 mungbean genotypes, showing their distribution across 11 chromosomes. Physical positions are indicated in megabase pairs (Mb), and SNP density is represented by a colour gradient ranging from dark green (low density, 1) to red (high density, 127) to reveal SNP distribution

A total of 35,49,948 raw SNPs were physically mapped with the *Vigna radiata* genome sequence serving as a reference [50]. Of these, 26,39,464 SNPs were assigned to 11 chromosomes, while 9,10,484 were located on non-chromosomal contigs. After applying filtering criteria, 4,307 high-quality SNPs were retained for genetic analysis of the 206 mungbean mini-core germplasm. These SNPs were unevenly distributed across the 11 mungbean chromosomes (Fig. 3). The average SNP count per chromosome was 392, with an average inter-SNP distance of 74.42 Kb (Supplementary Table S2).

### Population structure and phylogenetic analysis

**Fig. 4:**
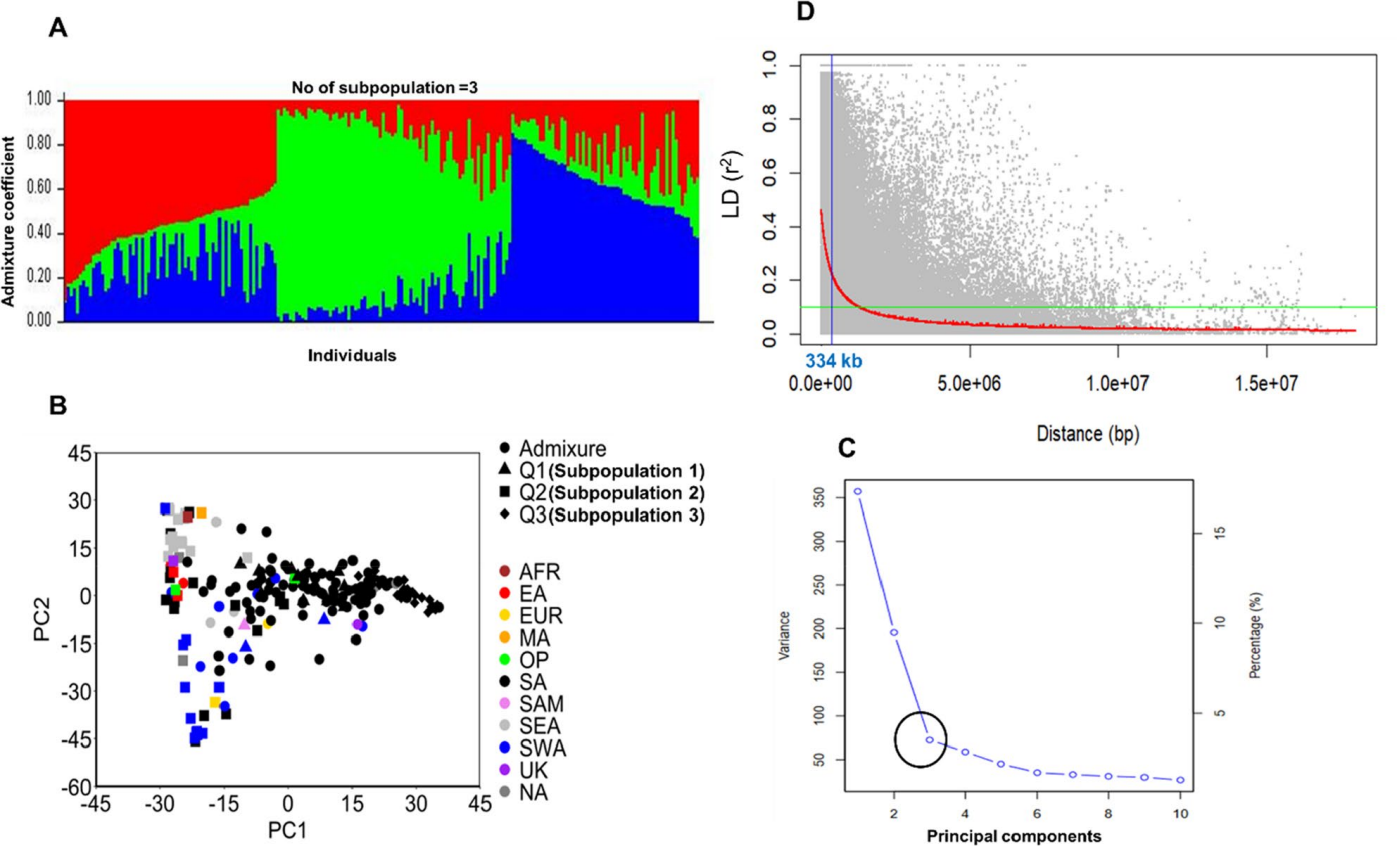
Population structure analysis and linkage disequilibrium (LD) of 206 mungbean genotypes based on 4,307 SNP markers. **A **Population classification using STRUCTURE 2.3.4, identifying three distinct subpopulations (K = 3) based on the second-order rate of change in the likelihood distribution. Subpopulation is indicated by three different colors- red, green and blue. **B** Principal Component Analysis (PCA) of 4307 SNPs showing the subpopulation (cluster coefficients ≥ 70%) with geographical origin. Different shapes represent population structure groups and colors represent geographical origin. AFR: Africa, EA: East Asia, EUR: Europe, MA: Central America, OP: Oceania and the Pacific, SA: South Asia, SAM: South America, SEA: Southeast Asia, SWA: Southwest Asia, UK: unknown, NA: North America. **C** Scree plot displaying the eigenvalues and the proportion of variance explained by each principal component. **D** LD decay analysis based on SNP of 206 mungbean germplasm genotypes. The curve represents the average LD decay across 11 chromosomes, with LD declining to r² = 0.1 at approximately 334 Kb

The STRUCTURE analysis of the current GWAS panel classified the germplasm into three distinct subgroups, comprising 15, 53, and 23 genotypes in subgroups I, II, and III, respectively (Fig. 4A), while the remaining 115 germplasm exhibited an admixed genetic background. Principal component and kinship analyses identified three distinct groups, aligning with the sub-populations detected by the Structure analysis (Fig. 4B). In the PCA, the first two principal components accounted for 28.36% of the total variation observed. Although the number of genotypes varied across geographical regions, with some regions represented by only a few accessions, the majority (approximately two-thirds) of the genotypes originated from South Asian (SA) germplasm. These SA genotypes were distributed across all inferred subpopulations, with a higher concentration observed in subpopulations 1 and 3. In contrast, germplasms from Africa (AFR), East Asia (EA), Europe (EUR), North America (NA), Oceania Pacific (OP), Southeast Asia (SEA), and Southwest Asia (SWA) were primarily grouped within subpopulation 2. The scree plot illustrated a rapid decline in the variance explained after the first three PCs (Fig. 4C), with the elbows suggesting the presence of approximately three subpopulations (K = 3).

Genome-wide linkage disequilibrium (LD) analysis, based on an r² threshold of 0.1, revealed an LD decay distance of 334,493 bp (Fig. 4D), exceeding the average inter-SNP distance across all chromosomes. This indicated that the 4,307 filtered SNPs (MAF ≤ 0.05) provided sufficient resolution for GWAS in this study.

### Genome-wide association analysis of yield-related traits

**Fig. 5:**
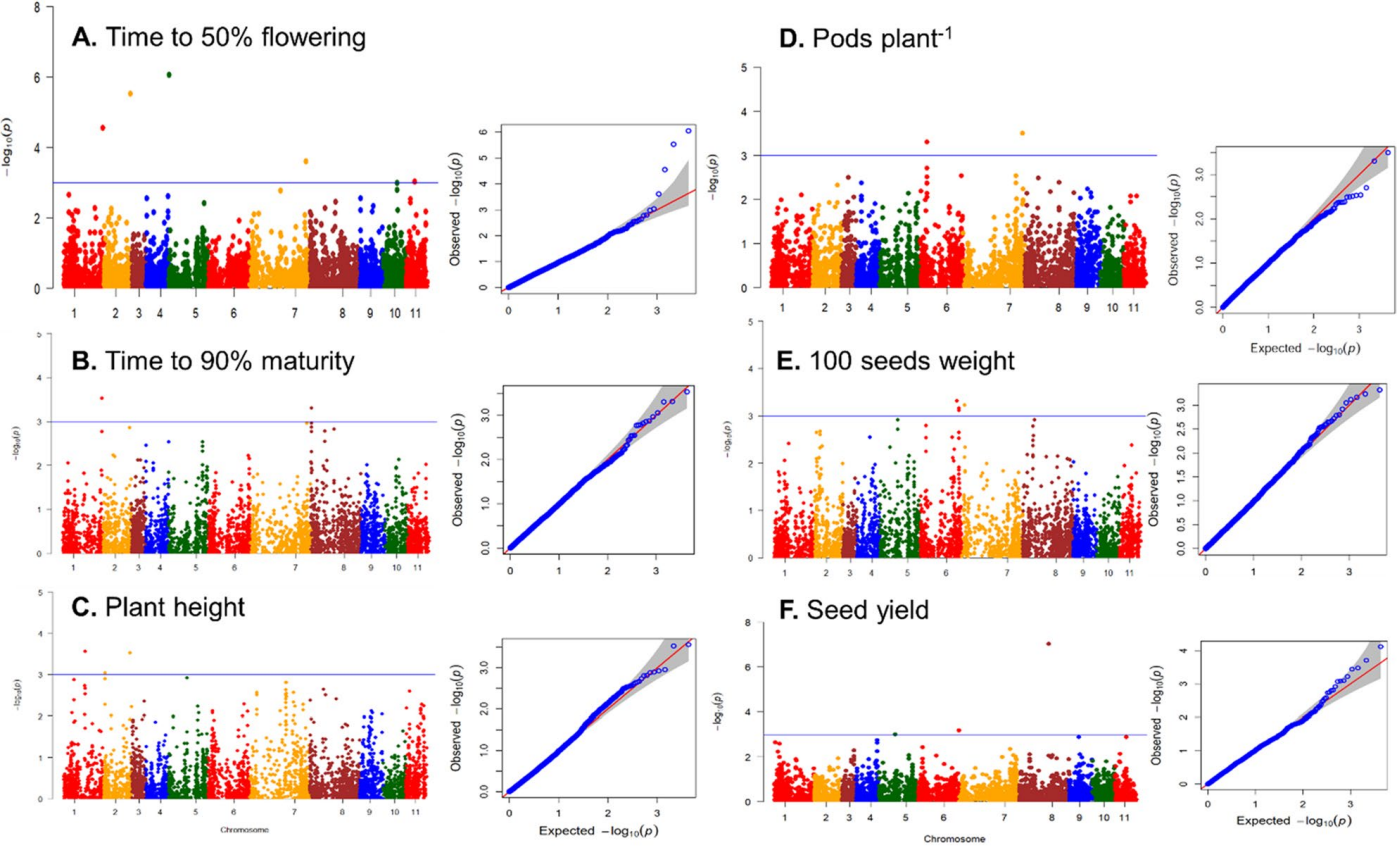
Manhattan plot and QQ plot of the six traits time to 50% flowering (**A**), time to 90% maturity (**B**), plant height (**C**), pods plant^− 1^ (**D**), 100 seeds weight (**E**) and seed yield (**F**). The x-axis indicates the SNP location along the 11 mungbean chromosomes and the y-axis represents -log10(p) for the p-value of the marker-trait association. The blue horizontal line indicates the significance threshold at p-values (-log_10_) above ≥ 3.00

**Fig. 6:**
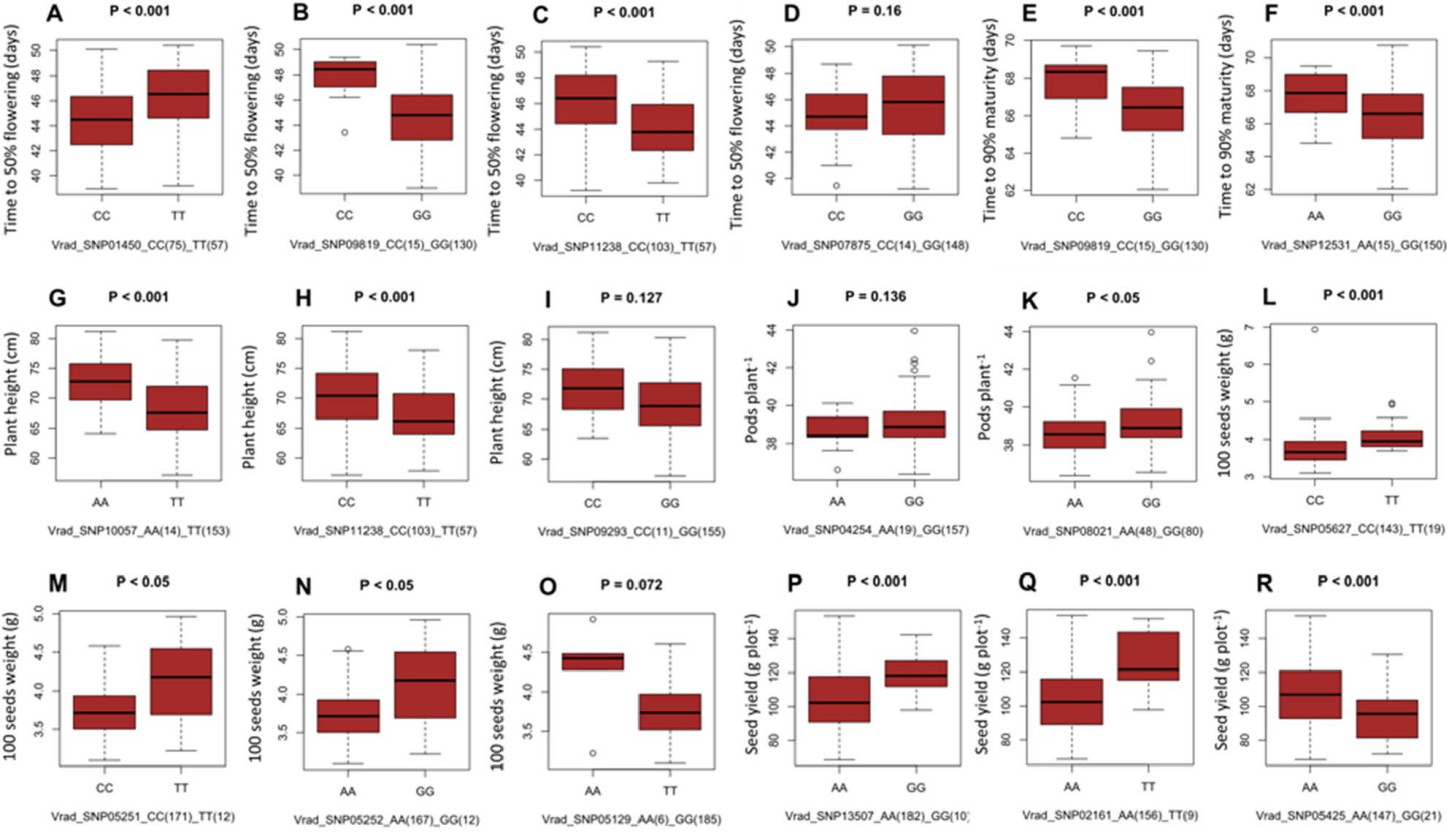
Phenotypic effects of the allelic groups for 18 significant SNP markers associated with the six traits in 206 mungbean genotypes. For each locus, genotypes were divided into two groups based on the allelic type. Significant differences between these two allele groups were evaluated using t-test (*P* < 0.05). Each boxplot represents the MET-derived BLUPs for each genotype. The number of genotypes harboring the corresponding allele is shown in parentheses. Panels (**A**–**D**) represent days to 50% flowering, (**E**–**F**) days to maturity, (**G**–**I**) plant height, (**J**–**K**) pods plant^-1^, (**L**–**O**) 100-seed weight, and (**P**–**R**) seed yield plant^-1^

The GWAS analysis was conducted using multi-year (MET) derived BLUPs of 206 genotypes for six traits, as they provide a more robust representation of overall genotypic performance across diverse environmental conditions. The GWAS results identified 18 significant SNPs (p-values of ≥ − log_10_ (3.00)) associated with the six traits, located in 16 genomic regions on chromosomes 1, 2, 5, 6, 7, and 8 (Fig. 5; Table 2). Additionally, assessing the allelic effects of the significant SNPs revealed that 15 markers caused a significant difference (P value < 0.05) in the respective traits between genotypes with the two allele groups (Fig. 6). Four SNPs associated with time to flowering were found on chromosomes 1, 2, 5, and 7, explaining 5–9% of the phenotypic variance for this trait (Fig. 5A; Table 2). For the SNP marker Vrad_SNP01450 on chromosome 5, genotypes with the CC allele exhibited a lower mean value than those with the TT allele (Fig. 6A). Conversely, genotypes with the CC allele showed higher mean values for SNP markers Vrad_SNP09819 on chromosome 1 and Vrad_SNP11238 on chromosome 2 compared to those with GG and TT alleles, respectively (Fig. 6B, C).

Regarding maturity, two significant SNPs were identified on chromosomes 1 and 8, and these markers explained 6–7% of the phenotypic variance for time to 90% pod maturity (Fig. 5B; Table 2). Genotypes carrying the CC allele for SNP marker Vrad_SNP09819 on chromosome 1 and the AA allele for SNP marker Vrad_SNP12531 on chromosome 8 took longer to mature compared to genotypes with the GG allele (Fig. 6E, F).

Three significant SNPs linked to plant height were located on chromosomes 1 and 2, each explaining 6% of the phenotypic variance for plant height (Fig. 5C; Table 2). Genotypes with the TT allele for SNP marker Vrad_SNP10057 and SNP marker Vrad_SNP11238 on chromosome 2 had shorter plant heights than those with the AA and CC alleles of the respective markers (Fig. 6G, H).

Two significant SNPs linked to pods plant^-1^ were identified on chromosomes 6 and 7, and these markers explained 4–5% of the phenotypic variation for pods plant^-1^ (Fig. 5D; Table 2). Genotypes with the GG allele for SNP marker Vrad_SNP08021 produced higher average pod counts than those with the AA allele (Fig. 6K).

Four significant SNPs associated with 100 seeds weight were found on chromosomes 6 and 7, and these markers explained 5–10% of the phenotypic variance for seed size (Fig. 5E; Table 2). The genotypes containing the TT allele of SNP marker Vrad_SNP05627 on chromosome 7 and SNP marker Vrad_SNP05251 on chromosome 6 were linked to a larger seed size than the CC allele (Fig. 6L, M). For SNP marker Vrad_SNP05252 on chromosome 6, the genotypes containing the GG allele had larger seed size than the genotypes with AA allele (Fig. 6N).

Three SNPs linked to seed yield were distributed on chromosomes 5, 6, and 8, and these markers explained 3–6% of the phenotypic variance for the seed yield (Fig. 5F; Table 2). Genotypes containing the GG allele of SNP marker Vrad_SNP13507 on chromosome 8 and the TT allele of SNP marker Vrad_SNP02161 on chromosome 5 had higher mean seed yield compared to the AA allele of the respective markers (Fig. 6P, Q). In contrast, genotypes carrying the AA allele for SNP marker Vrad_SNP05425 on chromosome 6 exhibited higher seed yield than those with the GG allele (Fig. 6R).

### Candidate genes

The nearest neighboring genes within the LD decay (334 kb) upstream and downstream of each significant SNP were examined for the positional candidate genes (Table 3). For time to flowering, four significant SNPs were mapped to genomic regions with annotated genes in mungbean, including genes with diverse functions such as pyruvate dehydrogenase E1 component subunit alpha-3 (chloroplastic), transporter, cycloartenol synthase, and adagio protein 3. For maturity, two significant SNPs were identified, one of which overlapped with a SNP associated with time to flowering. Three significant SNPs related to plant height were mapped, with two located near annotated genes and one in an uncharacterized region. Two significant SNPs associated with pods per plant were mapped to genomic regions, one of which was uncharacterized. Four candidate genes associated with seed weight were identified, all with functional annotations. Additionally, three genes were found near significant SNPs linked to yield, with one of these genes being functionally annotated.

**Table 3:** Candidate genes containing significant SNPs. Chromosome number (Chr), SNP position (Pos), allele, P.value, minor allele frequency (MAF), phenotypic variance explained (PVE), allele and functional annotation of the candidate genes

Traits	SNP ID	Chr	Pos	Allele	*P*.value	MAF	PVE (%)	Candidate Gene ID	Functional annotation
Time to 50% flowering	Vrad_SNP01450	5	431,635	C/T	8.94E-07	0.46	5	LOC106760161	pyruvate dehydrogenase E1 component subunit alpha-3, chloroplastic
	Vrad_SNP11238	2	23,895,929	C/T	2.95E-06	0.39	8	LOC111240615	probable polyol transporter 6
	Vrad_SNP09819	1	35,321,682	G/C	2.81E-05	0.22	9	LOC106762531	cycloartenol synthase
	Vrad_SNP07875	7	51,625,795	G/C	0.000245	0.17	6	LOC106765618	adagio protein 3
Time to 90% maturity	Vrad_SNP09819	1	35,321,682	G/C	0.000294	0.22	7	LOC106762531	cycloartenol synthase
	Vrad_SNP12531	8	419,595	G/A	0.000487	0.17	6	LOC106771237	protein IQ-DOMAIN 14
Plant height	Vrad_SNP09293	1	19,418,616	G/C	0.000273	0.15	6	LOC106758838	PXMP2/4 family protein 4
	Vrad_SNP11238	2	23,895,929	C/T	0.000295	0.39	6	LOC111240615	uncharacterized XR_002666069.1
	Vrad_SNP10057	2	1,415,113	T/A	0.001109	0.16	6	LOC106779365	Probable arabinosyltransferase ARAD1
Pods plant^− 1^	Vrad_SNP08021	7	53,158,029	G/A	0.000316	0.42	5	LOC106767886	uncharacterized XP_014508333.1
	Vrad_SNP04254	6	5,703,944	G/A	0.000491	0.17	4	LOC106764105	immediate early response 3-interacting protein
100 seeds weight	Vrad_SNP05129	6	32,887,306	T/A	0.000482	0.07	10	LOC106763732	L-type lectin-domain containing receptor kinase IX.1-like
	Vrad_SNP05627	7	3,086,302	C/T	0.000585	0.2	5	LOC106769090 LOC106767299	dehydrodolichyl diphosphate synthase 6
	Vrad_SNP05252	6	34,744,531	A/G	0.000685	0.12	9	LOC106763971	putative cyclin-A3-1
	Vrad_SNP05251	6	34,712,582	T/C	0.000764	0.11	10	LOC106762959	protein anthesis pomoting factor 1
Seed yield	Vrad_SNP13507	8	26,394,157	A/G	9.21E-08	0.08	4	LOC111242320	uncharacterized XP_022640703.1
	Vrad_SNP05425	6	36,829,198	A/G	0.000637	0.19	3	LOC106764013	uncharacterized XP_014503694.1
	Vrad_SNP02161	5	14,654,535	A/T	0.000955	0.14	6	LOC106762406	isoamylase 3, chloroplastic

### Distribution of favorable alleles and seed yield performance

**Fig. 7:**
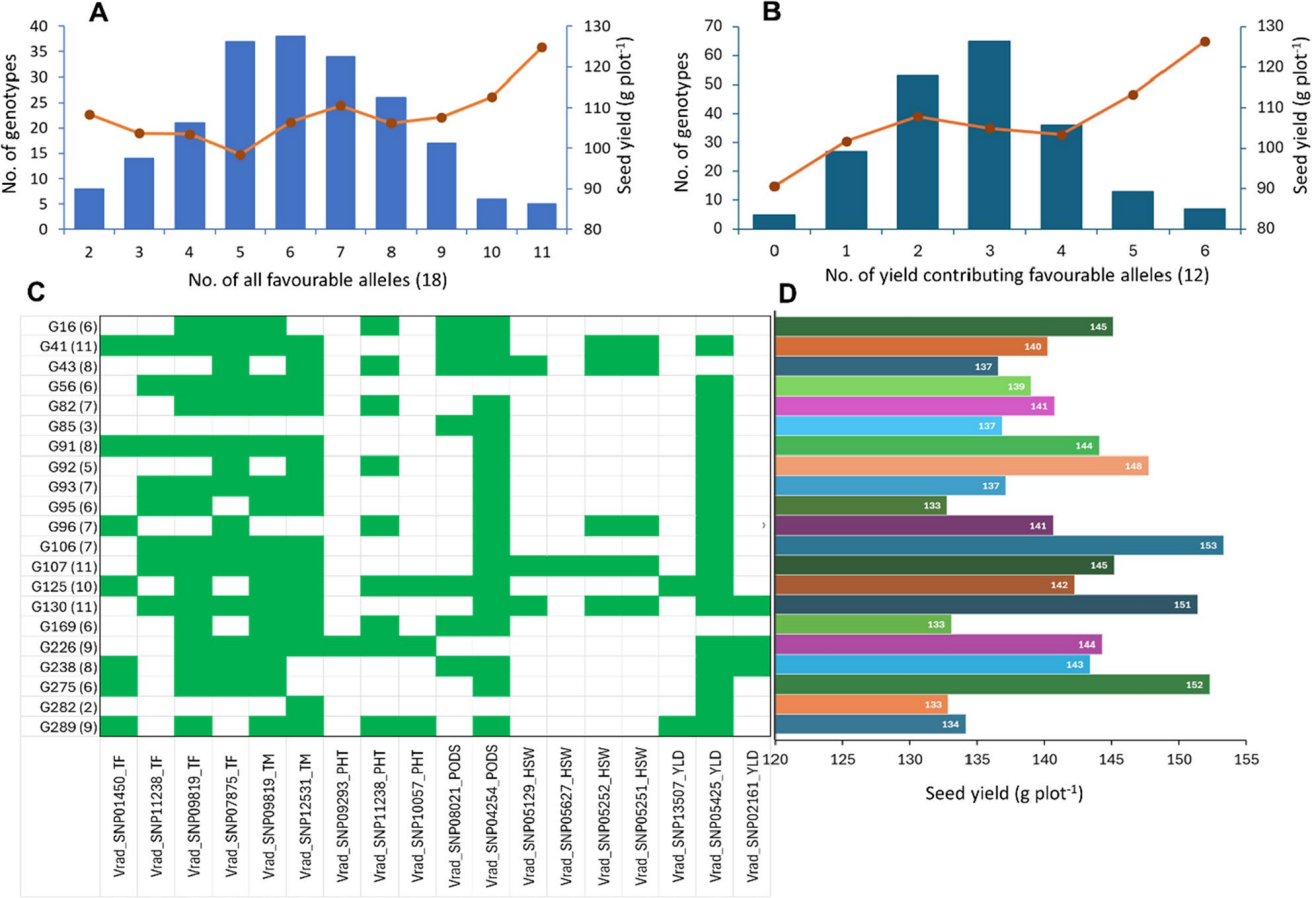
The number of genotypes and the mean BLUPs of seed yield with a varying number of favorable alleles for all 18 significant SNPs associated with the six traits (**A**) and only for the 12 SNPs associated with yield-related traits (plant height, pods plant^-1^, seed weight, and seed yield) (**B**). The bar graph represents the number of genotypes (primary Y-axis), and the line graph represents the mean BLUPs of seed yield (secondary Y-axis) of each group. Distribution of the 18 favorable alleles and corresponding yield performance in the 21 superior genotypes selected based on the highest seed yield from the combined MET analysis. The green heatmap illustrates the distribution of favorable alleles (**C**). In the heatmap, the number of favorable alleles for each genotype is shown in parentheses with the genotype name on the Y-axis and the SNP names with the associated traits on the X-axis. The histogram depicts the mean yield of the 21 superior genotypes (**D**), where different histogram colour represents the individual genotypes

To examine the cumulative effects of favorable alleles on yield performance, genotypes were grouped based on the total number of favorable alleles identified from the 18 significant SNPs associated with six traits (Fig. 7A). Most genotypes carried 5–7 favorable alleles, indicating a skewed distribution toward an intermediate accumulation of beneficial alleles. Fewer genotypes had either low (2–3) or high (10–11) numbers of favorable alleles, showing limited representation at both extremes. The mean BLUPs of seed yield displayed a positive trend, indicating that genotypes with a higher number of favorable alleles generally produced greater yields, suggesting additive and complementary effects of favorable loci on yield performance. Since phenological traits showed a negative correlation with yield-related traits, we further examined the combined effects of 12 SNPs associated with plant height, pods plant^− 1^, 100 seeds weight, and seed yield. Similarly, fewer genotypes had either low (0–1) or high (5–6) numbers of favorable alleles, with most containing 3–4. Seed yield increased steadily with the accumulation of favorable alleles, indicating that accumulating yield-enhancing alleles has a cumulative impact on overall productivity (Fig. 7B).

To identify superior genotypes with a high number of these favorable alleles, the distribution of the 18 favorable alleles among the top 10% high-yielding genotypes (21), selected from the multi-environment trial (MET) analysis, is shown in Fig. 7C, D. The number of favorable alleles in these 21 high-yielding genotypes ranged from 2 to 11, with an average of 7. Two genotypes, G41 and G107, along with G130, had the highest count of favorable alleles (11). The genotypes G125, G226, G289, G43, G91, and G238 possessed between 8 and 10 favorable alleles. Notably, genotypes G91, G106, G107, G125, and G130 are particularly interesting because they combine a higher number of favorable alleles with superior yield performance.

### Genomic prediction

**Fig. 8:**
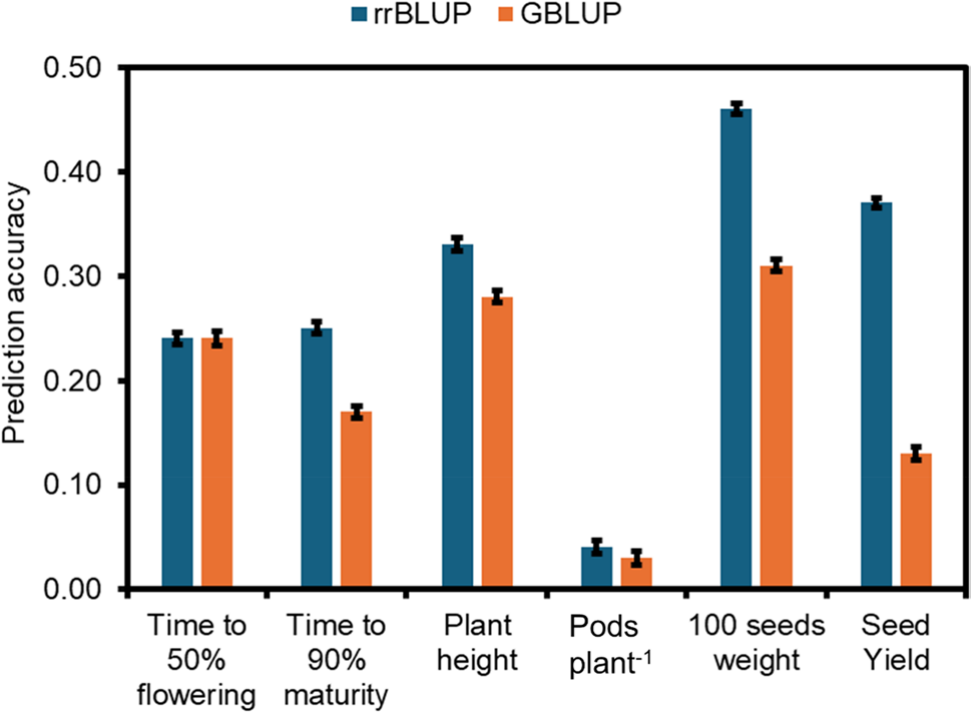
Genomic prediction accuracies for various traits in mungbean were evaluated using 4,307 SNP markers through gBLUP and rrBLUP approaches

The genome-wide prediction accuracy values obtained from the GBLUP and rrBLUP approaches for the studied yield-related traits are presented in Fig. 8. In the rrBLUP analysis using the full set of 4307 SNPs, the highest prediction accuracy was obtained for 100 seeds weight at 0.46, followed by seed yield (0.37) and plant height (0.33). The lowest accuracy was recorded for pods plant^− 1^ (0.04). Similarly, under the GBLUP approach, 100 seeds weight showed the highest prediction accuracy (0.31), followed by plant height (0.28), with the lowest accuracy also observed for pods plant^− 1^ (0.03).

## Discussion

Phenological and yield-related traits are key factors influencing seed yield and ultimately determining overall crop productivity. These traits also serve as key selection targets in plant breeding programs for improving the seed yield and phenological adaptation in the targeted environment. Therefore, germplasm collections are routinely evaluated for yield and yield-related traits in multiple environments to facilitate genetic improvement. The complex inheritance patterns of yield and strong environmental effects were reported for different legumes [12, 33]. Identifying the genetic mechanisms controlling these traits is essential for advancing the development of high-yielding mungbean cultivars. In this study, the evaluation of diverse mungbean mini-core germplasm across multiple years revealed substantial phenotypic variation for the phenological and yield-related traits. This study emphasized the importance of multi-environment evaluation to identify stable and high-performing genotypes for breeding programs targeting yield improvement under variable environmental conditions. This study identified several novel genomic regions associated with phenological and yield-related traits in mungbean. Moreover, a set of superior high-yielding genotypes harboring a greater number of favorable alleles was identified. Importantly, this study also provides evidence supporting the feasibility of genomic prediction for yield-related traits in mungbean.

The phenotypic data revealed significant variation among the 206 mungbean mini-core germplasm in Bangladesh conditions for key phenology and yield-related traits. The observed variability suggests the presence of substantial genetic diversity within the studied germplasm, which is essential for trait improvement through breeding. A moderate to high heritability was observed for most of the traits in the individual year, indicating that genetic factors predominantly govern these traits. However, the significant environmental (year) effects and genotype × year interaction and comparatively lower heritability for the combined multi-year (MET) analysis for most of the traits suggest possible complexity in their inheritance patterns, which may lead to difficulty in breeding efforts and highlight the necessity of multi-environment trials to identify stable and adaptable genotypes suitable for diverse agroecological conditions. Previous studies have also reported high narrow-sense heritability for pod length, pods plant^-1^, seed size, and pod yield [12, 52]. Traits with high heritability enable breeders to shorten breeding cycles, leading to faster genetic gains [12]. The violin plot distributions and PCA analyses further supported these findings, revealing clear year-to-year fluctuations in phenology, plant height, and yield traits, which likely resulted from environmental variability across years. For example, the lower rainfall in 2018 compared to the other two years resulted in shorter plant height and earlier maturity (Supplementary Table S3), highlighting how environmental fluctuations affect plant growth and development. Similarly, Dudley et al. [53] found that higher temperatures and lower rainfall affected flowering duration in mungbean. The approximately normal trait distributions in the MET data suggest that most traits are polygenic and quantitatively inherited, aligning with previous studies in mungbean and other legumes [25, 27, 53]. The PCA results also revealed the strong positive association between seed yield and pods plant^-1^, while flowering and maturity exhibited negative correlations with yield, indicating a trade-off between growth duration and reproductive output, which has been widely reported in legumes [54]. These results highlighted the importance of understanding genotype × environment interaction to identify stable and adaptable genotypes suitable for diverse agroecological conditions and also emphasized the need to identify genotypes having early maturity with superior yield performance under variable environments.

Mining favorable SNP alleles is essential for improving key phenological and yield-related traits in mungbean through marker-assisted selection (MAS). Among the various approaches, association mapping is particularly effective for identifying such alleles linked to complex traits. In this study, GWAS analyses were performed to dissect the genetic basis of phenology and yield-related traits and to identify the genomic regions carrying superior alleles for use in breeding programs. Considering the high genetic variation and strong genotype × environmental interactions observed in all traits, the combined multi-year (MET) dataset was used for GWAS to obtain robust associations. The success of GWAS studies for complex traits primarily relies on the population structure and the extent of linkage disequilibrium (LD) between functional alleles and adjacent SNP markers. The population structure analysis using principal component analysis (PCA) and model-based clustering (STRUCTURE) revealed distinct subpopulations within the mungbean panel, consistent with previous reports of genetic differentiation in mungbean mini-core germplasm [18, 38]. The stratification observed in the population highlights the importance of controlling population structure in GWAS to minimize spurious associations. Linkage disequilibrium (LD) analysis showed an average LD decay distance of 334 Kb and an average inter-marker distance of ~ 74 Kb, suggesting adequate genome coverage and sufficient resolution to detect QTL regions associated with complex traits. The GWAS study identified 18 significant SNPs associated with the six traits located in 16 genomic regions distributed on chromosomes 1, 2, 5, 6, 7 and 8, indicating the complex genetic regulation of mungbean phenology and yield-related traits, which corroborates the previous result of complex genetic basis of phenology and yield-related traits in mungbean [55, 56, 57]. We also conducted GWAS analyses for each year and did not identify any common, significant SNPs associated with the different traits, highlighting the strong environmental influence on these yield-related traits (data not shown). For phenological traits (time to 50% flowering and maturity) and plant height, we identified nine significant SNPs on chromosomes 1, 2, 5, 7, and 8. For time to 50% flowering, we identified a significant SNP (Vrad_SNP11238) in chromosome 2, which is located within 47 bp of a previously identified region (23895882 bp) associated with flowering time by Dudley et al. [53, 58] also identified a region of 164.87 kb (36.172–42.480 Mb) in chromosome 2 associated with flowering time in munbgean. This SNP (Vrad_SNP11238) was also associated with plant height, highlighting the pleiotropic effect of the candidate genes on these two traits. For yield-related traits, we identified nine novel genomic regions in chromosomes 6 and 7 for pod plant^− 1^ and 100 seeds weight, and in chromosomes 5, 6, and 8 for seed yield. Majunatha et al. [59] conducted GWAS analyses with 126 mungbean germplasm and found significant SNPs located in chromosome 1 for days to flowering, chromosome 7 for plant height, chromosome 11 for pods plant^− 1^, chromosome 8 and 9 for 100 seeds weight and chromosome 3 for seed yield. Sandhu and Singh [57] conducted a GWAS study in a USDA collection of 482 mungbean accessions and found significant SNPs on chromosomes 1, 3, and 5 for days to flowering, chromosome 1 for plant height, and chromosome 2 for seed size.

The identified significant SNPs accounted for only 3–10% of phenotypic variation, highlighting the complex quantitative nature of the traits, which are controlled by multiple small-effect loci and are largely influenced by environmental conditions. Similarly, the low-to-modest effects of the SNPs were reported for yield-related traits under optimum conditions [59] and under saline conditions [18]. Although the individual effects of each SNP were modest, their collective contribution is meaningful for breeding, as even small-effect alleles can provide cumulative gains when pyramided through marker-assisted or genomic selection strategies. We analyzed the average phenotypic effect of each allele group associated with the 18 significant SNPs and identified 15 favorable alleles linked to 6 traits, for which genotypes carrying contrasting alleles exhibited significant differences in MET-derived BLUP values. Most genotypes carried 5–7 beneficial alleles, while extremely low or high counts were rare. This pattern suggests that most genotypes in the population possess a moderate genetic advantage, which can be strategically exploited in breeding programs to accumulate additional favorable alleles through recurrent selection. The positive association between total favourable alleles and seed yield highlights the cumulative contribution of these loci and reinforces the value of pyramiding small-effect alleles to enhance productivity. A strong positive association was observed for the only yield-enhancing SNPs, in which seed yield increased steadily with increasing allele number. These results highlight the effectiveness of the favourable alleles for developing high-yielding mungbean varieties through marker-based gene pyramid strategies; however, the functional effects of these alleles require further validation. Previous studies have demonstrated the effectiveness of marker-based gene pyramid strategies [60, 61]. Distribution of these favourable alleles in the selected high-yielding 21 genotypes revealed that the genotype carried an average of seven favourable alleles, with few genotypes: (e.g., G41, G107, G130) possessing up to 11 alleles. Genotypes such as G91, G106, G107, G125, and G130 combined high allele counts with superior yield performance. Those genotypes would be of particular interest, as crossing them could help develop a cultivar with all the desired characters and high yield. The moderate genomic prediction accuracies observed for 100 seeds weight and seed yield further support the polygenic nature of these traits, with small-effect QTLs. These results align with previous research in soybean by Ravelombola et al. [62] using rrBLUP, and by Duhnen et al. [63] using gBLUP. Likewise, earlier studies in crops such as wheat [64], rice [65], and chickpea [34] reported moderate to high GP accuracies for yield-related traits with both models. In contrast, prediction accuracy for pod plant^− 1^ was extremely low, which is likely due to its low heritability in the MET analysis and strong environmental influence. The GP results from our study demonstrate the potential to accurately predict breeding values for key yield traits in mungbean at early generations, enabling faster genetic gains through shortened breeding cycles. However, traits that are strongly influenced by environmental variation may require more precise phenotyping, larger training populations, or environment-specific prediction models to improve prediction accuracy. Regarding candidate genes linked to significant SNPs, four key genes were associated with time to flowering: *pyruvate dehydrogenase E1 component subunit alpha-3 (chloroplastic)*, *polyol transporter 6 (PMT6)*, *cycloartenol synthase (CAS)*, and *adagio protein 3 (ADO3)*. Pyruvate dehydrogenase supports auxin-mediated organ development, and mutations in its mitochondrial E1 alpha subunit have been linked to organ defects, suggesting an indirect role in flowering [66]. PMT6 is part of the polyol/monosaccharide transporter family, functioning in pollen and young xylem cells, potentially linking it to reproductive development [67]. CAS catalyzes cycloartenol formation in sterol biosynthesis, critical for membrane integrity and plastid function. CAS1 mutations disrupt this pathway, impairing plastid biogenesis and development [68, 69]. ADO3, also known as FKF1, regulates circadian rhythm and photoperiodic flowering via blue-light sensing and protein degradation, promoting flowering under long-day conditions through interaction with GI and modulation of CO expression [70].

For time to pod maturity, *IQ-DOMAIN 14 (IQD14)*, a calmodulin-binding protein, plays a scaffolding role in microtubule-associated signaling and regulation of plant growth and development [71]. *ARAD1*, associated with plant height, encodes a glycosyltransferase essential for pectic arabinan biosynthesis. It modifies RG-I side chains in the cell wall, impacting cell expansion and plant structure [72]. Three genes were linked to 100 seeds weight: *LecRK-IX.1*, an L-type lectin receptor-like kinase involved in signal perception; *DPS6*, which participates in dolichol biosynthesis for protein glycosylation [73]; and *APRF1*, a WD40 repeat protein promoting flowering and contributing to embryo and endosperm development during seed formation. Finally, for seed yield, *ISA3* encodes a chloroplastic debranching enzyme involved in starch degradation. Though its role in energy metabolism is clear, its direct effect on seed yield remains uncertain and warrants further investigation [74]. The putative genes identified in the present study need further functional validation for their deployment in mungbean breeding programs.

These results emphasize the need to combine genomic tools with multi-environment and multi-trait phenotypic selection to improve trait-based breeding. A practical strategy would involve (i) using GWAS-identified markers to screen breeding materials for favorable alleles at early generations, (ii) applying genomic prediction models to estimate breeding values for complex yield traits, and (iii) advancing only those lines that combine high genomic estimated breeding values with stable performance across environments. Such an integrated approach would accelerate allele pyramiding, improve selection accuracy for polygenic traits, and enable the development of early-maturing, high-yielding mungbean cultivars with broader environmental adaptability. However, this study used only a moderate number of genotypes evaluated across three years in a single location and low-density SNP markers, which may limit mapping resolution, leading to the identification of only a few markers with modest phenotypic effects and candidate genes inferred solely by physical proximity, without functional validation. Therefore, future studies should use larger and more diverse panels, conduct multi-location trials, apply higher-density genotyping and fine mapping, validate genomic prediction in independent populations, and functionally confirm candidate genes to enhance breeding applicability.

## Conclusion

The comprehensive evaluation of 206 mungbean genotypes for phenology and yield-related traits over three years identified a few promising genotypes that will serve as valuable genetic resources for mungbean varieties with the potential to increase yield and productivity. The GWAS analysis led to the identification of several novel marker-trait associations (MTAs) and a few putative candidate genes. While the roles of these candidate genes in governing agronomically important traits require further functional validation, the identified MTAs offer valuable tools for selecting germplasm with favorable alleles. Moderate prediction accuracies for the 100 seeds weight and seed yield highlight the potential of utilizing GP for mungbean breeding. The insights gained from this study can facilitate the development of SNP-based molecular markers for traits of interest, thereby accelerating the mungbean breeding program and supporting the creation of improved ideotypes. Future studies should prioritize multi-location validation of MTAs and candidate genes, expand training populations for genomic prediction, and integrate functional genomics to enhance the reliability and practical impact of genomics-assisted mungbean improvement.

## Supplementary Information

Supplementary Material 1.

## Data Availability

The datasets generated during and/or analysed during the current study are available either in the manuscript or can be available from the corresponding author upon reasonable request.
